# Brinjal leaf diseases detection based on discrete Shearlet transform and Deep Convolutional Neural Network

**DOI:** 10.1371/journal.pone.0284021

**Published:** 2023-04-05

**Authors:** S. Abisha, A. M Mutawa, Murugappan Murugappan, Saravanan Krishnan

**Affiliations:** 1 Department of Electronics and Communication Engineering, Rohini College of Engineering and Technology, Nagercoil, India; 2 Computer Engineering Department, College of Engineering and Petroleum, Kuwait University, Kuwait, Kuwait City, Kuwait; 3 Department of Electronics and Communication Engineering, Kuwait College of Science and Technology, Doha, Kuwait; 4 Department of Electronics and Communication Engineering, School of Engineering, Vels Institute of Sciences, Technology, and Advanced Studies, Chennai, Tamilnadu, India; 5 Center of Excellence for Unmanned Aerial Systems (CoEUAS), Universiti Malaysia Perlis, Arau, Perlis, Malaysia; 6 Department of Computer Science Engineering, Anna University Regional Campus, Tirunelveli, Tamilnadu, India; Bennett University, INDIA

## Abstract

Different diseases are observed in vegetables, fruits, cereals, and commercial crops by farmers and agricultural experts. Nonetheless, this evaluation process is time-consuming, and initial symptoms are primarily visible at microscopic levels, limiting the possibility of an accurate diagnosis. This paper proposes an innovative method for identifying and classifying infected brinjal leaves using Deep Convolutional Neural Networks (DCNN) and Radial Basis Feed Forward Neural Networks (RBFNN). We collected 1100 images of brinjal leaf disease that were caused by five different species (*Pseudomonas solanacearum*, *Cercospora solani*, *Alternaria melongenea*, *Pythium aphanidermatum*, and Tobacco Mosaic Virus) and 400 images of healthy leaves from India’s agricultural form. First, the original plant leaf is preprocessed by a Gaussian filter to reduce the noise and improve the quality of the image through image enhancement. A segmentation method based on expectation and maximization (EM) is then utilized to segment the leaf’s-diseased regions. Next, the discrete Shearlet transform is used to extract the main features of the images such as texture, color, and structure, which are then merged to produce vectors. Lastly, DCNN and RBFNN are used to classify brinjal leaves based on their disease types. The DCNN achieved a mean accuracy of 93.30% (with fusion) and 76.70% (without fusion) compared to the RBFNN (82%—without fusion, 87%—with fusion) in classifying leaf diseases.

## 1. Introduction

Agriculture is the backbone of the world economy and is vital to the socio-economic structure of many countries, including India. Trees and plants are essential to preserving the earth’s ecosystem and atmosphere for a healthy environment. In plants, leaves are flat, thin, flattened lateral structures that serve as principal agents of photosynthesis and transpiration. Leaf inspections are the most effective method of detecting plant diseases/deficiencies. The farmers used to check the plants at regular intervals, and if they didn’t recognize illness symptoms, they found it difficult to apply fertilizer or pesticide. Crop disease detection and recognition are crucial areas of research in agriculture. Detecting leaf/plant disease could be useful for monitoring large areas of crops and detecting symptoms even while they are still hidden within leaves. Farmers will be unable to detect actual disease deficiencies across a vast area, affecting the soil and plants. If detected earlier, the crop’s quality can be preserved, and the problem can be minimized. This allows for early detection of nutritional shortages, illnesses, and pests across a large region by monitoring plant health. It is always more difficult to produce high-quality agricultural products when a disease cannot be predicted early, resulting in significant crop losses.

Therefore, detecting and assessing crop diseases is essential to ensuring the quality of crops [1]. Developing an intelligent leaf disease diagnosis system also involves identifying and recognizing weeds in a farming area. It is more difficult to identify weeds in vegetable plantations than in crop plantations because of the uneven spacing between the plants. A number of researchers are developing low-cost, computationally efficient weed deduction systems based on machine learning and deep learning algorithms. A deep learning (DL) method is usually preferred over a machine learning method. The DL does not require separate feature extraction and feature selection modules to identify and classify weeds. It is therefore crucial to develop a fast, efficient, low-cost, and robust method for detecting crop disease outbreaks [2].

In developing countries, the lack of information about specific plant diseases complicates the control of various types of plant diseases. A study by Abang et al. found that farmers in Cameroon, Africa, are capable of identifying 21% and 16% of plant diseases and pests, respectively. An examination of farmers’ knowledge of several illnesses revealed a lack of understanding of biotic stress in crops and viral illness management [3]. Consequently, farmers should have sufficient training and hands-on experience in identifying and controlling diseases. It is, however, impossible to manage such a vast area without substantial expertise and facilities. Recent advances in low-cost smartphones have opened the possibility of detecting diseases from photographs of foliage affected by the disease [2]. Cameras on smartphones function as vision sensors, collecting color information in the red, green, and blue spectrums. Based on the visual representation of the leaves, Wang et al. have used these data in conjunction with machine learning algorithms to recognize disease patterns [4]. Mathematical functions have been traditionally used to describe symptoms and to examine their correlation with different disease patterns. In order to identify different diseases, these handcrafted characteristics/features are fed into machine learning algorithms such as Artificial Neural Networks (ANN), Support Vector Machines (SVM), and others, and the most effective features that result in improved accuracy are used [4–6]. Recent advances in computing are enabling researchers to extract deep features from plant leaves to detect diseases and infections. Deep Learning (DL) algorithms are being used to accomplish this task. DL methods, however, require large-size data to produce a more generalized solution and may not provide higher accuracy when training with fewer samples/data. Convolutional Neural Networks (CNN) are primarily used for disease identification in the literature [7–10].

Various crops like tomato leaves, grape leaves, corn leaves, cherry leaves, and lemon leaves were investigated for identifying different types of diseases. Machine learning techniques have been used for disease detection and classification following the development of deep learning techniques, which have the potential to increase accuracy significantly. Due to the economical nature of brinjal leaves, they are cultivated extensively in Tamil Nadu, India and can be used for studying specific diseases [11, 12]. Moreover, brinjal leaf diseases are always harder to identify through visual inspection than other leaf diseases, owing to the fact that environmental changes have always affected brinjal leaves.

The following are the major contributions to the brinjal leaf disease diagnosis system:

- A novel approach based on the hybrid method of FCM and EM segmented images is proposed to investigate its effectiveness even with low-quality images.
- A DST-based fusion image with multiresolution analysis is used for extracting color, texture, and structural features for improving neural network performance even for low-quality images, especially for detecting brinjal plant diseases.
- We used Deep Convolutional Neural Network (DCNN) and Radial Basis Feed Forward Neural Network (RBFNN) models to identify specific diseases present in brinjal leaves.

The rest of the paper is organized as follows: Section 2 discusses related works on brinjal leaf disease diagnosis. A description of the proposed methodology can be found in section 3. Detailed descriptions of the experimental results are presented in section 4. The conclusion of the paper is presented in section 5.

## 2. State-of-the-art works related to leaf disease detection

In this section, we describe the latest work on detecting leaf diseases using machine learning, deep learning, and hybrid methods.

### (a) Handcrafted features and machine learning methods in leaf disease detection

Using morphological methods, Concepcion II et al. [13] have extracted different regions from lettuce leaves and classified them using machine learning algorithms such as Linear Discriminant Analysis (LDA), Support Vector Machine (SVM), and Artificial Neural Network (ANN) to predict plant growth and diseases. According to Massi et al., serial and hybrid classifiers were employed to classify six types of leaf diseases and damages based on three types of features, including color, shape, and texture [14]. An image-based multilayer perceptron and support vector machine-based system for diagnosing crop diseases has recently been developed by Kurmi et al.. using leaf images from tomato, potato, and pepper plants. Color features were extracted from the leaves and different gaussian order functions were used to discriminate the leaf diseases of different leaves. The maximum mean classification rate achieved with SVM was 94.35% [5].

In [15], the authors have classified the four different types of defects (surface, morphological, color, and black mould) with normal fruit using histogram and gray-level co-occurrence matrix (GLCM) features. These features are classified into five classes using Radial Basis Probabilistic Neural Network (RBPNN) and achieved a maximum mean classification rate of 88%. Almadhor et al. have proposed a novel AI framework to identify three different types of diseases (dot, rust, canker, and mummification) in the Guava plants using handpicked features from high-resolution images [16]. Here, they have extracted color and texture features from the leaf disease images and classified them using five machine-learning algorithms. A maximum mean accuracy of 99% is achieved using the Bagged tree (BT) classifier using color features compared to the other classifiers (KNN, SVM, Complex Tree, and BT). Fuzzy C-Means clustering-based segmentation is used to segment the diseased location in corn leaves [17]. They have used the Plant Village image dataset for developing their model to identify three different types of diseases in corn leaf such as blight, gray spot, rust, and normal leaf. Maximum mean accuracy of 96.81% is achieved using FCM compared to 80.60% and 80.25% using K Means clustering and Otsu’s segmentation methods, respectively.

### (b) Deep learning methods in leaf disease detection

Using deep neural networks, Sun et al. developed a novel fusion algorithm for detecting maize leaf blight and obtained a maximum precision rate of 91.83% [18]. Convolutional neural networks (CNNs) were mostly employed for categorizing healthy leaves in order to detect and analyze leaf sicknesses [19]. Based on the open-source database (87848 images), Konstantinos Ferentinos has developed different deep neural network models (AlexNet, AlexNetOWTBn, GoogleNet, Overfeat, and VGG) for 58 classes of plants and 25 plant species and has achieved a maximum mean detection rate of 99.53% on unseen images [7] According to Mohanty et al., AlexNet and GoogleLeNet were used to identify the leaf diseases of 14 crop spices (mostly apple, blueberry, cherry, grape, orange, pepper, etc.) based on color, grayscale, and segmented images, with an accuracy of 99.35%. Compared to the traditional methods of weed detection [20], Jin et al. presented a CenterNet deep neural network model that achieved a maximum precision rate of 95.6%. The model focused mainly on identifying plants rather than analyzing multiple species. This technique can also increase plant identification accuracy and performance by lowering the number of trained datasets and even the complexity of weed identification [21].

Zhou et al. proposed a restructured residual dense network (RRDN) for the detection of tomato leaf diseases. A hybrid deep learning model combines a deep residual network with a deep dense network. As a result, the proposed method used fewer training parameters, was computationally efficient, and reported a maximum mean detection rate of 95% [8]. To detect four different types of diseases (Mites, Black rot, Leaf blight, and Black measles) that affect grape leaves, Xie.et al. developed a graph of leaf diseases data-based and devised a real-time leaf disease detection algorithm based on DR-IACNN [9]. They reported a maximum precision rate of 81.1% with a detection speed of 15 frames per second. Using DNNs such as VGG16, VGG19, GoogleNet, ResNet101, and DenseNet201, Krishnaswamy et al. were able to distinguish ten types of leaf diseases (Tobacco Mosaic Virus, Little Leaf Disease, Epilachna beetle, Two-spotted spider mite, Cercospora leaf spot, Brown spot, Citrus Hindu mite, Citrus canker, yellow vein mosaic virus, and Leaf hopper) from each other [11]. A maximum mean classification rate of 97.3% and 90% is reported by GoogleNet and VGG16, respectively. Later, the same researchers proposed an ensemble model based on VGG16 and SVM to classify the different types of diseases in Eggplant and achieved a maximum mean classification rate of 99.4% [12].

Anagnostis et al. have employed FFT to extract features from Anthrocnose fungal disease leaves and classified the diseases with DNN (VGG16, DensNet121, ResNet50, Inception-v3, customized CNN). In the end, their customized CNN achieved the highest disease recognition rate of 99% compared to other DNNs [22]. CNN has been used to detect fifteen types of diseases in three major crops (tomato, potato, and pepper). They achieved a maximum detection rate of 98% using different optimizer functions in CNN [23]. According to Guan et al., four types of DNNs (VGG19, VGG16, Inception-v3, and ResNet50) have been developed to classify apple plants’ disease severity levels. Using the VGG16 network, a maximum classification rate of 90.9% was achieved for classifying leaf diseases into healthy, early, middle, and end-stage [24]. Recently, Poornima et al. developed explainable vision transformer-based CNNs to diagnose three plant diseases: apple, maize, and rice. Their maximum disease recognition rates were 93.55%, 92.59%, and 98.33%, respectively [25].

The authors have proposed a modified MobileNetV2-based deep neural network to classify the lower-quality Cassava leaf images through data augmentation and Chebyshev orthogonal function-based convolution with leaf image histograms [26]. They have achieved a maximum mean classification rate of 97.7% without augmentation and 99.7% with Chebyshev orthogonal functions-based convolution. In [27], the authors have considered the leaf diseases of nine species namely, apple, cherry, corn, grape, peach, pepper bell, potato, strawberry, and tomato, and utilized transfer learning based DenseNet121 deep neural networks for identifying various types of leaf diseases. The maximum mean accuracy of 99.25% and 98.87% is achieved with 50 epochs, and 30 epochs, respectively using Plant Village Dataset. Finally, the best model with optimal hyperparameters has been deployed into the mobile operating system for designing a mobile app to detect the different types of diseases in real-time. In [28], the researchers have proposed a novel deep fusion stream architecture (CNN-SVM, CNN-Softmax) based inference model to identify nine different types of diseases (aphids, bacterial wilt, cercospora melongenae, collar rot, Colorado potato beetle, little leaf, spider mites, Phomopsis blight, tobacco mosaic virus) in Eggplant using two different datasets and achieved a maximum mean classification rate of 98.9% compared to the state-of-the-art methods (Inception V3, VGG19, MobileNet, NasNetMobile, VGG16, and ResNet50). A novel 14-layer DCNN architecture has been proposed to classify 42 different types of leaf diseases from 12 plants using a large dataset of images (139,000) [29]. The proposed architecture achieved a maximum mean classification rate of 99.65% compared to the state-of-the-art models such as AlexNet (96.60%), Inception-V3Net (98.3%), ResNet50 (98.30%), and VGG16 Net (98.4%).

### (c) Hybrid methods in leaf disease detection

Recently, Attada et al. have proposed both brinjal disease classification and prediction system using SVM and CNN, respectively, and achieved a maximum mean classification rate of 98.1% using the proposed method compared to the state-of-the-art methods (Rider-Cuckoo Search Algorithm (87.77%), Ensemble model (96.33%), Probabilistic Programming approach (93.02%), Internet of Agro-Things (97.76%), and GMDA- Logistic model (98.01%) [30]. However, the authors have not clearly mentioned the dataset used and the number of images used for training, testing, and the diseases classified/predicted by their proposed model.

Recently, Ashutosh et al. have proposed two different methodologies (deep features and hand-crafted features-based leaf disease classification) to classify the leaf diseases in apple, corn, potato, tomato, and rice plants spices [31]. Here, CNN is used to extract the deep features and these features are used to classify the leaf diseases using Bayesian optimized SVM classifier and achieved a maximum mean accuracy of 92.2%. However, the hand-crafted features extracted using color moments, Histogram of Gradient, and GLCM with PSO-based optimization and random forest classifier reported a maximum mean classification rate of 96.1%.

In the above state-of-the-art works on leaf disease detection, the researchers have mostly applied deep learning models to large data sets with a variety of methods in order to identify leaf diseases in a variety of spices (tomato, bell pepper, eggplant, apple, guava, etc.). There have been very few studies that used machine learning algorithms or hybrid approaches, and most of the earlier studies used open-source databases to train the models and analyze complete or full leaf images. Also, we could not find a notable number of works based on leaf disease classification using segmentation (disease localization) and fusion approaches in the literature. The use of Deep Learning models with different methodologies for classifying diseases in various crops has been demonstrated in a few studies. The images in the image dataset were directly fed into deep neural networks for disease classification in earlier works. In the literature, very few studies have been conducted on disease segmentation and classification. There are still a few vital crops for which there is no open-source dataset available. The importance of brinjal (Solanum melongena) has been overlooked in earlier works, and this crop has been produced in different countries and plays a crucial role in providing sufficient nutrients for humans. There have been several diseases affecting this crop. Among them, the five most common types of diseases are, (i) *Pseudomonas solanacearum*, (ii) *Cercospora solani*, (iii) *Alternaria melongenea*, (iv) *Pythium aphanidermatum* and (v) *Tobacco Mosaic Virus*. A total of six classes of brinjal leaves were collected in this study, including healthy leaves and leaves with the five diseases mentioned above. The diseased areas in the leaves are identified and segmented using FCM and EM, and multi-resolution information is extracted using the DST method. To identify leaf diseases, three features are extracted: color, texture, and structural features. These three features are fed into DCNN and RFNN classifiers to classify leaf diseases. Compared to our earlier work on brinjal leaf disease classification, the proposed methodology has shown good performance.

## 3. Proposed methodology

This section discusses the proposed methodology of brinjal leaf disease detection using DCNN.

### 3.1 Experimental settings and data description

**Fig 1:**
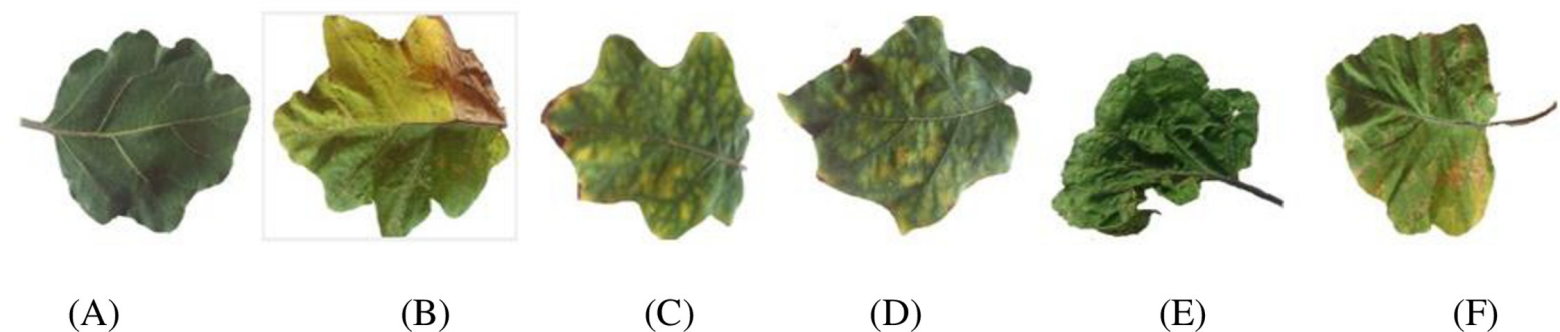
(A) Normal leaf (B) *Pseudomonas solanacearum*, (C) *Cercospora solani*, (D) *Alternaria melongenea*, (E) *Pythium aphanidermatum* and (F) *Tobacco Mosaic Virus*.

Among the various types of diseases that affect eggplant, five are the most common, and they have a significant impact on the brinjal leaf. In India, brinjal leaves were collected from different farms located in the northern part (Hosur) and southern part (Panakudi, Valliyoor, and Nagercoil) of Tamil Nadu, and Kerala (Kollam, Neyyattinkara). Brinjal leaf images were acquired in a natural environment under adequate natural lighting in the agricultural form (outdoor temperature: 29°C, no rainfall during data acquisition, humidity: 82%, wind speed: 70 kph). Most of the leaf images’ backgrounds consist of other overlapping leaves of the same plant or other plants, weeds, and soil. There was also a variation in the ambiance of the images within the same category. An expert at the Agricultural College and Research Institute in Killikulam, Tamilnadu, examined the leaves and categorized them into five major types of diseases using the microscopic examination. Sample images are shown in Fig 1 along with the number of images in each class as described in Table 1. The brinjal images are manually cropped and extracted as a single-leaf image; the white background is set to enhance the clarity of the image.

**Table 1:** Details of leaf database.

S.No	Types of leaf disease	Total no of images	Classes
1.	Healthy leaves (Normal)	400	**Healthy (400)**
2.	Pseudomonas solanacearum	220	**Infected (1100)**
3.	Cercospora solani	220	
4.	Alterneria melongenea	230	
5.	Pythium aphanidermatum	150	
6.	Tobacco Mosaic Virus	280	

**Fig 2:**
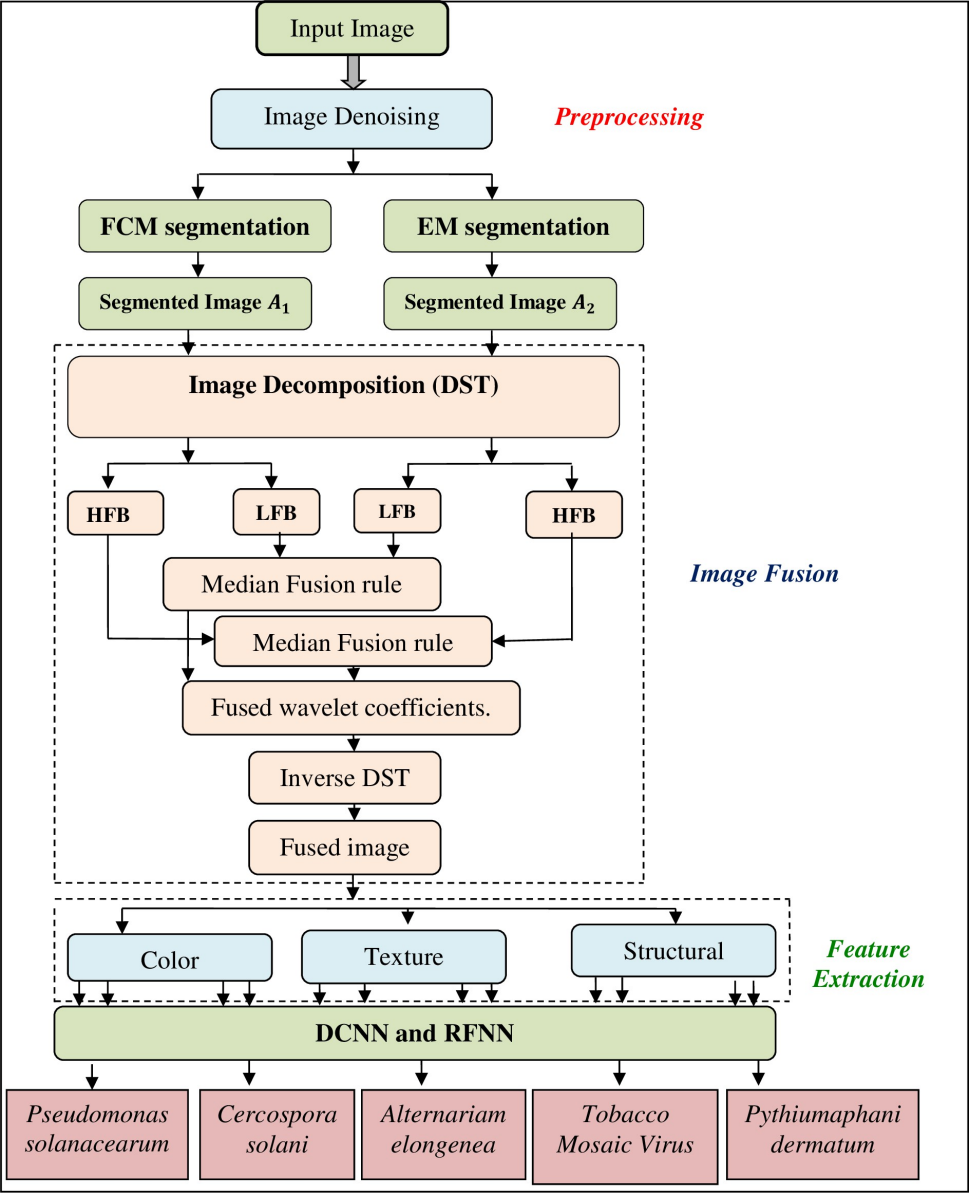
The proposed methodology to diagnose the brinjal leaf diseases.

We have captured the leaf images using the Nikon Digital camera Z5 kit with Nikon Z 24–200 mm f/4-6.3 lens in this study. The resolution of the images is 3840 × 2160 pixels. Images captured by the camera are stored in a memory and then transferred to a personal computer for processing. The computing system used for identifying leaf diseases is powered by Intel core i7-12700H processor and 32GB of RAM. To implement the image processing algorithm and deep learning algorithm for the detection of leaf diseases, MATLAB 2020a software is used. Fig 2 illustrates the steps involved in detecting brinjal leaf disease: 1) Preprocessing 2) Image Segmentation 3) Image Fusion 4) Feature Extraction, and 5) Classification. Leaf images are preprocessed to remove noise from brinjal leaf images after they are collected from brinjal leaves. According to Fig 2, there are five phases involved in diagnosing leaf diseases.

### 3.2 Image preprocessing

Preprocessing leaf images is essential before extracting meaningful information about leaf diseases. This work uses a Nikon camera to capture images of leaves, which are saved in RGB format. In this case, Gaussian noise affects the leaves primarily due to the high amplification applied to the blue color passage compared to green and red. Preprocessing is performed on the input data in three stages to improve its quality. Firstly, the filtered images are useful for identifying diseases because they remove Gaussian noise or white noise present in leaves by varying the Wiener filter’s noise density level.

The general equation for the wiener filter by restored image *R*(*u*, *v*) is given below,

R(u,v)=W(u,v).Rr(u,v) (1)

Here, *Rr*(*u*, *v*) is the received image, *W*(*u*, *v*) is the image spectrum for the wiener filter. It is many employed for the linearization of the blurred affected image [32]. In addition, it prevents sharp edges and high smoothing even in the veins of the leaf. Secondly, image enhancement is achieved by using the spatial domain method. This method enhances the original leaf image by sharpening and brightening it. This increases the contrast of the brinjal leaf image. Moreover, it prevents a sharp boundary and high smoothing even in leaf veins. As a final step, the images are resized to standard 256×256 pixels dimensions to reduce computational complexity.

### 3.3 Image segmentation

Image segmentation methods are used to segment preprocessed leaf images into smaller parts. This study uses the unsupervised image segmentation methods Fuzzy C Means (FCM) clustering and Expectation Maximization (EM) segmentation to segment the color images [6].

#### 3.3.1 Fuzzy C Means segmentation (FCM)

The FCM clustering technique involves one piece of data representing two or more clusters by minimizing the weighted sum of the squared error objective function with different membership values ranging from 0 to 1. Using this FCM algorithm, each data point is mapped to each cluster center according to its distance from the cluster center. In contrast to supervised segmentation, FCM-based segmentation is completely unsupervised, and it completely eliminates noisy spots. Moreover, no prior information is required for segmenting the infected parts of the leaf and there are no distortions after segmentation [14].

#### 3.3.2 Expectation Maximization (EM) method

The Expectation Maximization method uses a model approach to determine the local maximum region by applying the maximum likelihood estimate parameter in an iterative manner. During the convergence process of Expectation Maximization algorithms, multiple initial parameters are used to find the local minimum. Due to this, the algorithm becomes more stable, robust, fast, and easy to implement for any statistical model with incomplete data. The EM algorithm follows a process that alternates between an expectation step and a maximization step, unlike other concave functions-based optimization algorithms. Based on a parametric distribution, such as a Gaussian Mixture Model (GMM), each region in EM can be mathematically represented.

*3.3.2.1 Gaussian Mixture Model (GMM)*. Gaussian Mixture Model is a density-based model with a large number of component distributions. These distributions are used to represent a specific region to provide a multimodal density-based image. All points of data are considered as one-dimensional vector measurement in the preprocessed image *X* = {*x*_1_, *x*_2_,…., *x*_*j*_, …., *x*_*n*_}, the Gaussian density is calculated using the parameters such as means, and covariance matrix is given by:

$$(x_{j}/\varphi_{k})=\frac{1}{{\left(2\pi \right)}^{\frac{n}{2}}{\left|\varepsilon_{k}\right|}^{\frac{1}{2}}}\;exp\left(\frac{-1}{2}(x_{j}-{\bar{\mu }}_{k})\varepsilon_{k^{-1}}(x-{\bar{\mu }}_{k})\right)$$ (2)

Where ${\bar{\mu }}_{k}$ is the mean, $\varepsilon_{k^{-1}}(x-{\bar{\mu }}_{k})$ is the covariance matrix.

In the Gaussian mixture-based model, the probability distribution density for the leaf is given in Eq (3),

$$p(x_{j}/\theta )=\sum_{k=1}^{k}a_{k}(x_{j}|\varphi_{k})$$ (3)

Where *a*_*k*_ is the mixture weight based on the prior probability of the leaf image. If the Gaussian components are too low, then the variability of the images are unable to handle. In contrast, if the K value is too large, the GMM final model will be too complex. In our work, GMM is used with the help of the density-based model and achieved good results at *K* = 10 Gaussian components.

### 3.4 Discrete Shearlet transform based image fusion

The most common multi-resolution analysis (MRA) method using wavelet transformation (WT) is more efficient and powerful in detecting only pointwise singularities in the input data. Nevertheless, it does not analyze other types of singularities in data input, such as edges in clinical (MRIs, CTs, x-rays, etc.) and non-clinical (leaf images). In particular, it fails to analyze the different types of singularities in multi-dimensional data. In order to resolve this issue, the MRA method, which is based on the principle of directional orientation, can be applied. Shearlet transform is very powerful for analyzing the locations, scales, and orientations of singularities among the different types of MRA methods, such as wavelet transform, contourlets, complex wavelets, curvelets, and others. Thus, Shearlet transform produces a notable degree of accuracy in detecting edges in input data compared to other methods. Leaf-segmented images are processed using DST to extract features. The discrete Shearlet Transform is one of the most computationally efficient methods for performing multiresolution analysis (MRA) on a multidimensional signal. In addition to performing directional transformations at multiscale levels [33], it extracts features from different levels of DST transformation on an image.

**Fig 3:**
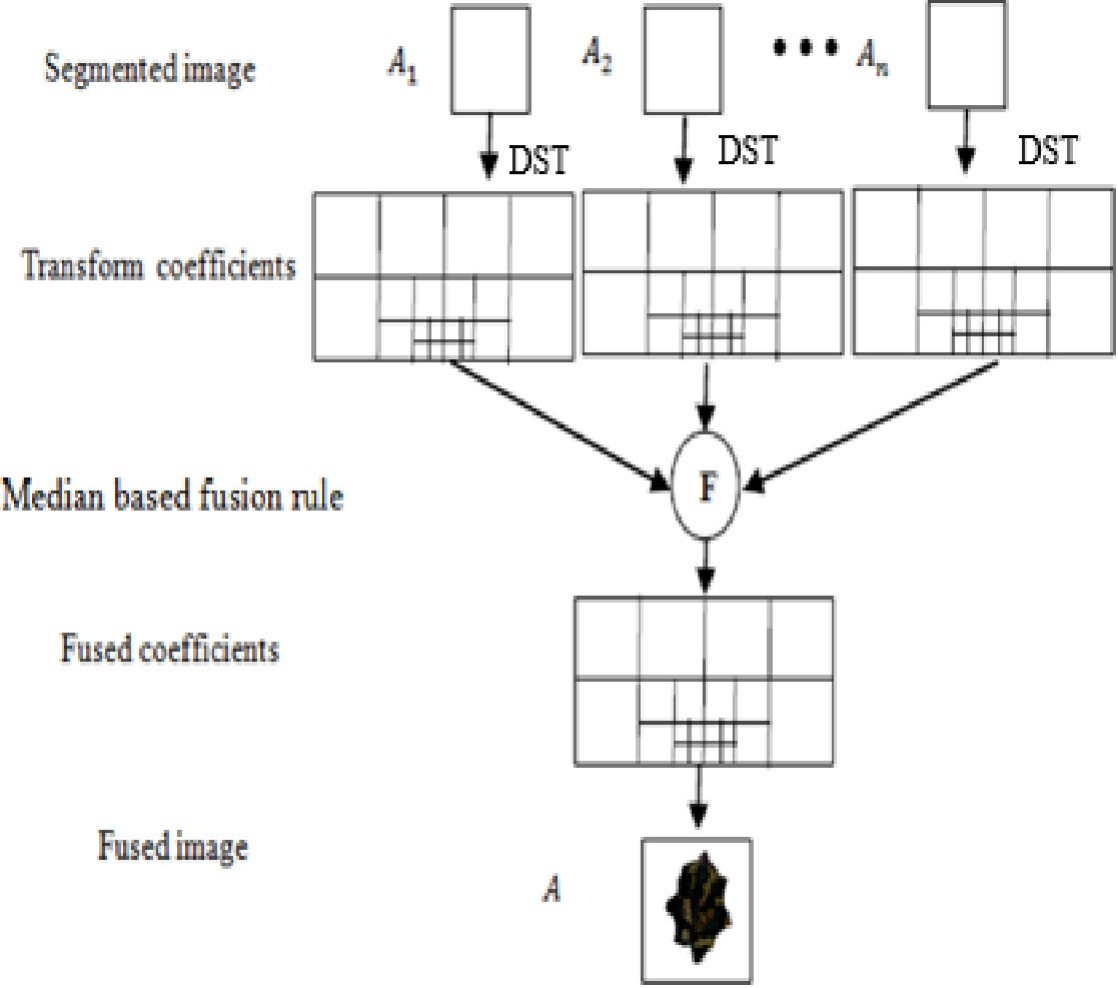
Image fusion based on discrete Shearlet transform.

In the DST, Shearlet parameters *a*, shear parameters *S*, and translation parameters are combined to achieve optimal sparse approximations [4]. Analysis of the above-mentioned elements yields the optimal sparse approximation *φ*_*x*_: *a* > 0, *S* ∈ *γ*, *tr* ∈ *γ*^2^. Fig 3 shows an image fusion model based on DST. Shearlet uses 4×4 decomposition and multi-resolution by applying the pyramid transformation. A feature vector is then computed from the decomposed image of a brinjal leaf.

The matrix *M*_*a*_ can be written as

$$M_{a}=S_{a}P_{a}$$ (4)

Where, *S*_*a*_ is the matrix shear and *P*_*x*_ is the parabolic related to scaling matrix.

$$S_{a}=\;\left(\begin{array}{l} 1\;s\\ 0\;1 \end{array}\right)$$ (5)

and

$$P_{a}=\;\left(\begin{array}{l} b\;0\\ 0\;\sqrt{b} \end{array}\right)$$ (6)

The function based on Shearlet Transform is

$$ST=\;\langle f,\varphi_{x}\rangle$$ (7)

The first step in DST is to decompose the segmented image by using a pyramid transform to produce different coefficients depending on the scales and directions. Following the medium fusion rule, these coefficients are fused using an inverse discrete Shearlet transform. To calculate the coefficient of median value, a 3 × 3 window is taken from the coefficient. To determine the median, these coefficients are sorted numerically, and the middle value is taken. The DST coefficient having greater absolute difference is estimated from the median value. In the same way, all the coefficients of the fused leaf image are achieved.

### 3.5 Image classification

The Radial Basis Function Neural Network (RBFNN) and Deep Convolutional Neural Network (DCNN) models are demonstrated for the classification of various types of diseases. This neural network receives input from the fused image based on many features, including Contrast, Correlation, Energy, Homogeneity, Mean, Standard Deviation, Skewness, Asymmetry, Border, Irregularity, Color, and Diameter.

#### 3.5.1 Radial Basis Function Neural Network (RBFNN)

**Fig 4:**
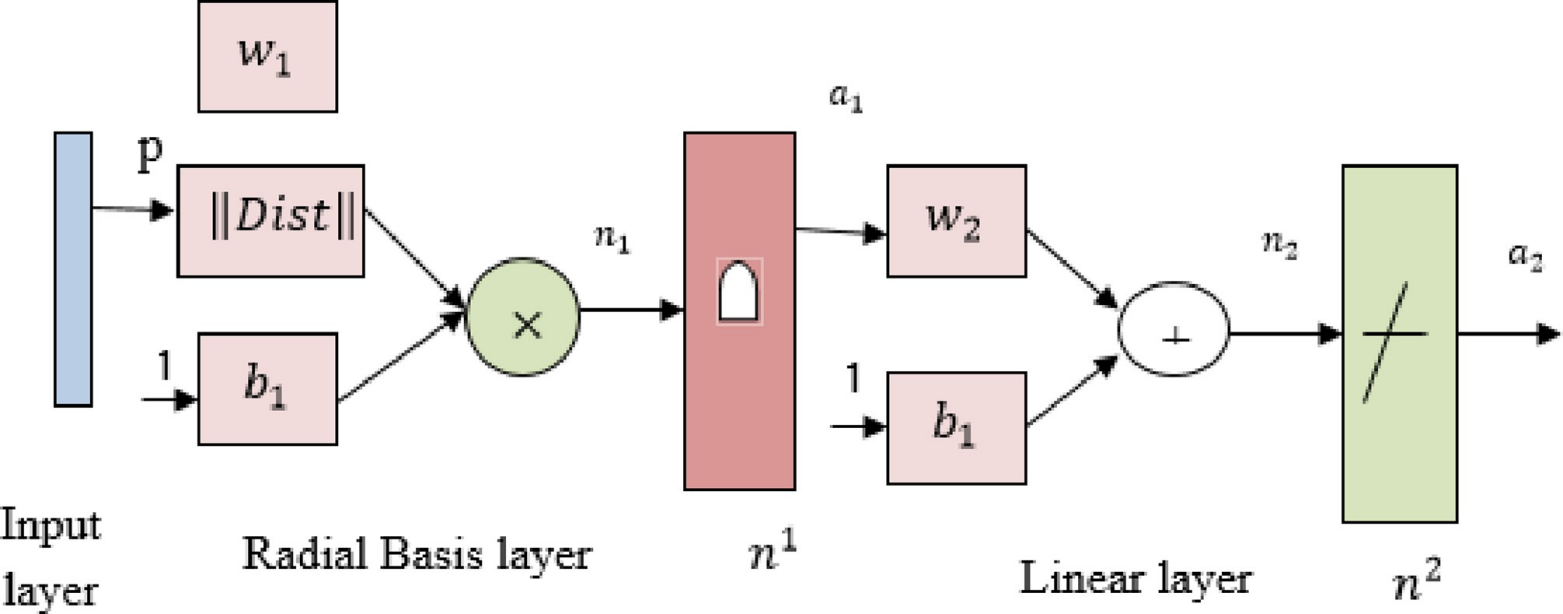
Structure of Radial Basis Function Neural Network.

Radial Basis Function Neural Network consists of three layers such as input layer, hidden radial basis layer of neurons, and output linear layer of neurons. Each node is connected to the previous neighborhood layer. The input variables are assigned based on the features taken from the fused images to each node. The hidden nodes are activated by the transfer function of Radial Basis function (RBF). In RBFNN, the number of hidden nodes based on the activation function is shown in Fig 4.

The input layer is operated with each neuron produced an input weight equal to the distance between input and weighted vector observed as ||*Dist*||. Each input neurons are element to element product by its input weight and its bias. Output layers are operated based on activation functions. During network training, parameters associated with activation functions are optimized based on the actual training features value. During the training phase, the linear combination of RBF’s is assigned to be error free when the training vector is considered accurate or non-stochastic. Radial Basis Functions are fitted using distance weighted regression. An RBF is represented by the same number of basis functions for each training point. Furthermore, each input neuron serves as the center of each basis function. The width of each kernel must account for the training points to maintain smooth operation.

#### 3.5.2 Deep Convolutional Neural Network (DCNN)

Deep Convolutional Neural Network for image recognition is based on each neuron arranged by overlapping regions in the feature vector. In deep learning, CNN is very robust and sensitive to each feature present in the leaf images. The typical CNN has several layers starting with a) convolutional level b) max pooling level c) Optimizer level d) Dropout level e) Completely connected level, and f) Classification level. We tested several types of DCNN with different number of layers. We utilized different number of layers in DCNN, finally, the eight-layer DCNN architecture gives the best accuracy in classifying the leaf diseases. Hence, we have proposed an eight-layer CNN for brinjal leaf disease classification. The result from the completely connected layer has 5-way SoftMax function for five classes (leaf diseases). Then, three consecutive convolution layers tracked by a maxpool layer are used to make the feature clearer and increase the accuracy of the classification. Each convolutional and fully connected layer is followed by a ReLU layer to make the training phase of the infected area faster. The input image was resized to the dimension of 256×256 (RGB) and convolved with 3 × 3 convolutional filter and dropout layers, and then it is given as the input to the fully connected layer with 1500 neurons. Then, these 1500 neurons are used to make predictions using SoftMax functions. The convolution operation is carried out with two functions and considered real number as the arguments. The convolution operation is defined as the

c(t)=∫y(a)z(t−a)da (8)

In deep learning models, the input is taken as a multidimensional vector where *I* is the input image and *K* is the two-dimensional kernel. The discrete operation for the convolution can be represented in terms of the mathematical expression as

$$s(i,j)=\;(I\;*\;K)(i,j)=\sum_{m}\sum_{n}I(i-w,j-u)K(w,u)$$ (9)

In this work, we have utilized AlexNet for classifying the leaf diseases. Because this network has been trained with larger size image data of different classes. The first five layers are used for convolutional operation and the remaining three layers are connected at the end of the neural network for the classification of different types of diseases. Table 2. shows parameter described in Deep Convolution Neural Network

**Table 2:** Parameter used in DCNN for training.

Parameter	Range of values	Optimal value
Number of layers	4–10	8
Number of inputs	1500	1500
Number of outputs	6	6
Activation function	Sigmoid, Tanh, SoftMax	SoftMax
Convolution filter size	3 × 3	3 × 3
Number of epochs	30–50	50
Learning rate	0.01–0.0001	0.001

Our present work has been carried out to fine-tune the parameters of the DCNN by performing an ablation study to fine-tune the hyperparameters used in the DCNN. In this case, we keep the number of kernels as a fixed size of 3×3. We tune the other hyperparameters such as the learning rate, momentum factor, activation function, and the number of convolutional layers according to the need. Furthermore, routing numbers are used to find accurate coupling coefficients for the routing numbers. The accuracy of routing will increase with an increase in the routing number. Several parameters were tested to determine the performance of the DCNN architecture. To perform cross-validation on each training dataset, a 10-fold cross-validation procedure was adopted. The best set of parameters were selected and applied following the achievement of very high accuracy and has been used to test the leaf dataset. Training will stop if the error in the validation set is less than the error in the previous iteration. The optimal hyperparameters will have been derived.

## 4. Results and discussion

**Fig 5:**
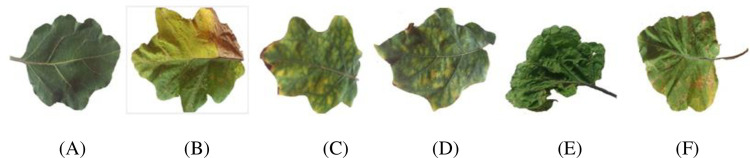
(A) Normal leaf (B) *Pesudomonas solanacearum* (C) *Cercospora solani* (D) *Alternaria melongenea* (E) *Pythium aphanidermatum* and (F) Tobacco Mosaic Virus.

For this study, we collected 1500 leaf images from the states of Tamilnadu and Kerala in India. Fig 5 shows images of diseases caused by bacteria, fungi, and viruses. The original images were then captured at a higher resolution, and we resized them first to 256×256 for a faster computation by reducing its pixels resolution.

**Fig 6:**
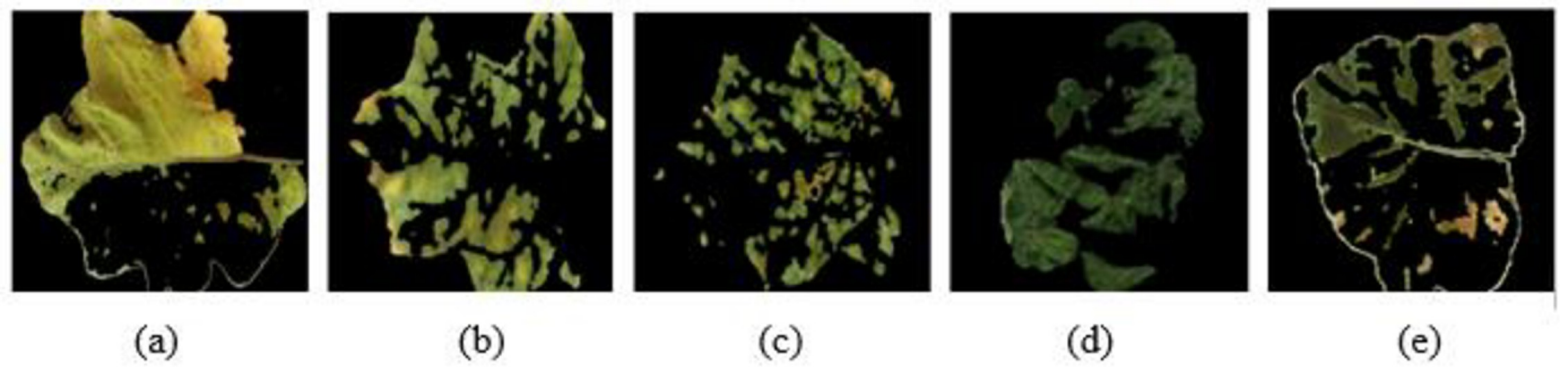
Fuzzy C Means clustering for brinjal leaf (a) *Pseudomonas solanacearum* (b) *Cercospora solani* (c) *Alternaria melongenea* (d) *Pythium aphanidermatum* (e) *Tobacco Mosaic Virus*.

**Fig 7:**
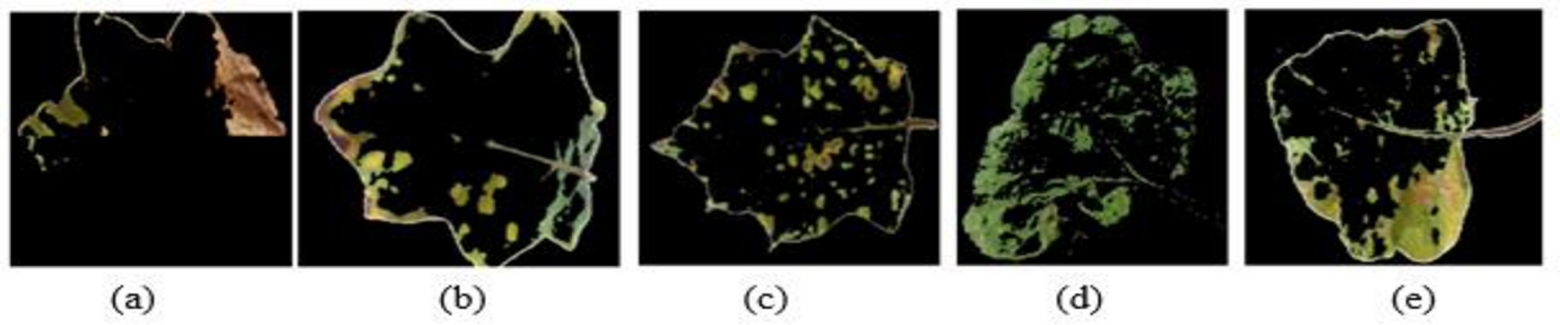
Expectation Maximization segmentation for brinjal leaf (a) *Pseudomonas solanacearum* (b) *Cercospora solani* (c) *Alternaria melongenea* (d) *Pythium aphanidermatum* (e) *Tobacco Mosaic Virus*.

To detect the presence of a diseases (discontinuity) and to extract edge with good orientation for retaining the essential information regarding the shapes of infected part of the leaf, the preprocessed images are then subjected to FCM clustering algorithm and EM segmentation for *Pesudomonas solanacearum*, *Cercospora solani*, *Alternaria melongenea*, *Pythium aphanidermatum* and *Tobacco Mosaic Virus* as shown in Figs 6 and 7.

**Fig 8:**
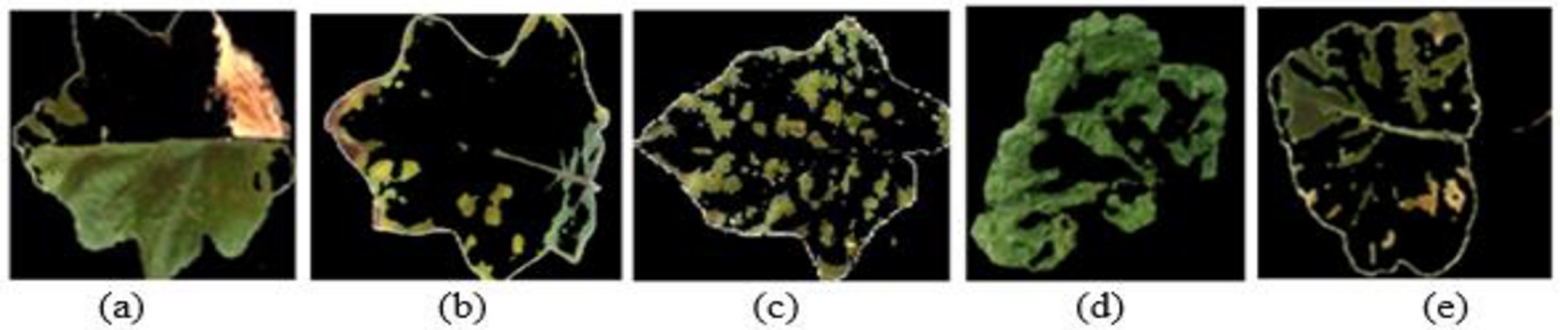
Image fusion based on Discrete Shearlet Transform (a) *Pseudomonas solanacearum* (b) *Cercospora solani* (c) *Alternaria melongenea* (d) *Pythium aphanidermatum* (e) *Tobacco Mosaic Virus*.

The Discrete Shearlet Transform (DST) provides better visual quality than other transformations [33]. It is due to the fact that discrete wavelet transforms (DWT) suffer due to artifacts, reduced contrast, and blurring. As illustrated in Fig 8, discrete Shearlet Transform-based fusion methods provide high visual quality in images.

The quality of the fused images was analyzed by considering the performance matrices such as Peak Signal to Noise Ratio (PSNR) and Structural Similarity Index Measure (SSIM). PSNR is a measure of image quality based on pixel difference between the segmented image and fused image is given by,

$$PSNR=10\;\log\frac{e^{2}}{MSE}$$ (10)

where,

$$MSE=\frac{1}{AB}\sum_{a=0}^{A-1}\sum_{b=0}^{B-1}e{\left(a,b\right)}^{2}$$ (11)

Structural Similarity Index Measure (SSIM) is a way for predicting, perceived quality of fused infected image which depends upon the computation of local mean and standard deviation are computed by using the formula as given below:

$$SSIM=\frac{\left(2\mu_{A}\mu_{B})(2\sigma_{A}\sigma_{B}\right)}{\left(\mu_{A}^{2}\mu_{B}^{2})(\sigma_{A}^{2}\sigma_{B}^{2}\right)}$$ (12)

where the mean value can be calculated by using this formula

$$\mu_{A}=\frac{1}{N}\sum_{i=0}^{N}A_{i}$$ (13)

$$\mu_{B}=\frac{1}{N}\sum_{i=0}^{N}B_{i}$$ (14)

Where the standard deviation value can be calculated by using this formula

$$\sigma_{A}=\sqrt[{2}]{\frac{1}{N}\sum_{i=1}^{N}{\left(A_{i}-\mu_{A}\right)}^{2}}$$ (15)

$$\sigma_{B}=\sqrt[{2}]{\frac{1}{N}\sum_{i=1}^{N}{\left(B_{i}-\mu_{B}\right)}^{2}}$$ (16)

It is pertinent to note that the structural similarity index measures improved when there is more dissimilarity between the fused image and the segmented image. Therefore, SSIM increased when original images were affectedly segmented. Consequently, PSNR is used to measure the quality of the fused image. This implies that the fused image has a level of clarity similar to that of the segmented image with clear vision. PSNRs and SSIMs for various images are shown in Table 3.

**Table 3:** PSNR and SSIM for leaf image.

Diseases	PSNR			SSIM		
	FCM	EM	Fused image	FCM	EM	Fused image
*Pseudomonas solanacearum*	11.2	20.28	**32.56**	0.54	0.62	**0.72**
*Cercosporasolani*	11.5	20.1	**37.45**	0.6	0.65	**0.78**
*Alternariamelongenea*	10.96	20.7	**32.89**	0.52	0.63	**0.73**
*Pythiumaphanidermatum*	10.98	20.54	**38.02**	0.59	0.65	**0.81**
*Tobacco Mosaic Virus*	10.32	19.54	**40.72**	0.5	0.69	**0.79**

The different types of features like structural, color and texture features are taken from the segmented leaf images and the fused images. Table 4 represents the value of various set of features extracted from the image before and after fusion. The following 4 features are computed from the leaf images to assess the performance of DST: (i) Contrast (ii) Correlation (iii) Energy, and (iv) homogeneity.

**Table 4:** Texture features of brinjal leaf images before and after fusion using DST.

Features	Before fusion				
	Pseudomonas solanacearum	Cercosporasolani	Alternaria melongenea	Pythium aphanidermatum	Tobacco Mosaic Virus
Contrast	0. 623	0.492	0.478	0.467	0.582
Correlation	0.9937	0.9884	0.9899	0.9912	0.9913
Energy	0.489	0.4467	0.4823	0.4345	0.4812
Homogeneity	0.9634	0.9824	0.9825	0.9832	0.9801
**After fusion**					
Contrast	0.683	0.583	0.517	0.692	0.613
Correlation	0.9912	0.9956	0.9924	0.9634	0.9934
Energy	0.562	0.724	0.475	0.527	0.573
Homogeneity	0.992	0.999	0.943	0.967	0.972

In Table 4, it can be seen that contrast features provide a low value for the leaf with *Pseudomonas solanacearum* disease and a value greater than ’1’ for the remaining diseases. A low correlation value is found for the leaf images with *Cercosporasolani*, whereas a high correlation value is found for the leaf images with *Pythiumaphanidermatum* disease. The value of homogeneity is low for the images with *Alterneriamelongenea* and the images with *Tobacco Mosaic Virus* in terms of energy consumption for leaf images taken with these two species. A color-based feature extraction procedure is then performed on the RGB leaf images [6] to obtain color-based features such as mean, standard deviation, and skewness based on the color-based features extracted in the RGB leaf images (Table 5).

**Table 5:** Color features of brinjal leaf images before and after fusion using DST.

Methods	Before fusion		
	Mean	Standard Deviation	Skewness
*Pseudomonas solanacearum*	126.34	68.23	-1.34
*Cercospora solani*	136.34	66.34	-1.18
*Alterneria melongenea*	143.23	65.24	-0.92
*Tobacco Mosaic Virus*	136.23	68.34	-0.64
*Pythium aphanidermatum*	124.67	81.34	-0.26
**After fusion**			
*Pseudomonas solanacearum*	142.78	70.67	-0.54
*Cercospor asolani*	154.23	69.56	-0.63
*Alterneria melongenea*	163.76	70.23	-0.34
*Tobacco Mosaic Virus*	154.76	73.67	-0.21
*Pythium aphanidermatum*	185.67	84.56	-0.14

From Table 5, it is noticed that the color features obtained from the DST fusion image gives better results when compared to segmented results also the clarity is improved when the skewness value is decreased, and standard deviation is increased in the fusion image. The feature based on structural data gives relevant information related to scope of the infected region of the leaf. Structural features (asymmetry, border irregularity, color, and diameter) are obtained for fused image [6] and segmented image as shown in Table 6.

**Table 6:** Structural features based on DST.

Features	Before fusion				
	*Pseudomonas solanacearum*	*Cercospora solani*	*Alternaria melongenea*	*Pythium aphanidermatum*	Tobacco Mosaic Virus
**Asymmetry**	4.32	2.79	2.46	7.33	3.67
**Border Irregularity**	0.18	0.31	0.15	0.25	0.64
**Color**	1.75	1.95	1.79	2.59	2.87
**Diameter**	5.79	3.55	7.67	4.36	3.46
**After fusion**					
**Asymmetry**	6.79	4.68	5.35	5.87	5.79
**Border Irregularity**	0.08	0.07	0.06	0.09	0.11
**Color**	2.65	2.52	1.35	3.58	2.46
**Diameter**	6.34	4.25	8.35	5.87	4.85

From the experimental results, it is highly evident that the DST based fusion methods show better results and it shows notable improvement in the spectral and spatial information compared with other wavelet transforms in leaf disease detection. Finally, we have used the feature vector that contains combined features such as textural, color and structural features to classify the leaf diseases and normal using Deep Neural Networks (DNN). These combined features are given as input to the neural network for automatic leaf disease detection. The network was trained with 75% of the images and the remaining 25% of images are used for testing. Table 7. shows the distribution of images into training and testing.

**Table 7:** Distribution of leaf images used in classification.

S.No	Types of leaf disease	Total no of images	Training Images	Testing Images
1.	Healthy leaves (Normal)	400	300	100
2.	Pseudomonas solanacearum	220	165	55
3.	Cercospora solani	220	165	55
4.	Alterneria melongenea	230	172	58
5.	Pythium aphanidermatum	150	112	38
6.	Tobacco Mosaic Virus	280	210	70

**Fig 9:**
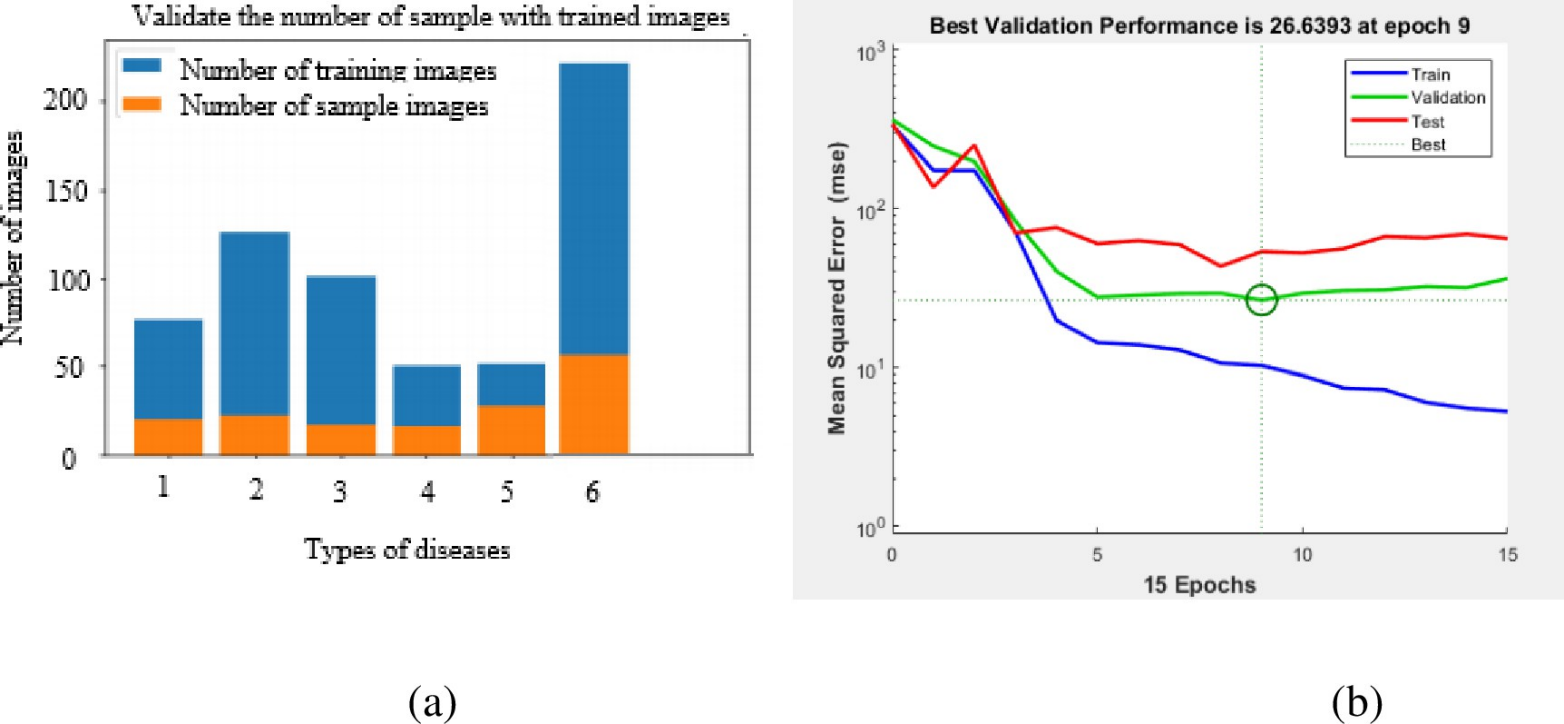
(a) Validate the number of samples with trained images (b) Best validation performance.

As Fig 9A shows the number of samples in validation process during its training phase and Fig 9B shows the best validation performance matrix in the deep convolutional neural network is shown below.

The validation error is maximum at 9^th^ epoch, hence, the training phase is stopped at that point and used weight and bias for further modeling. The performance of the DNNs in identifying the leaf diseases was assessed through four performance measures namely, sensitivity, specificity, precision, and accuracy.


$$Sensitivity=\frac{TP}{\left(TP+FP\right)}\cdot$$ (17)

$$Specificity=\frac{TN}{\left(TN+FP\right)}\cdot$$ (18)

$$Precision=\frac{FP}{\left(TN+FP\right)}\cdot$$ 19

$$Accuracy=\frac{TP+TN}{\left(TP+FP+TN+FN\right)}$$ 20

**Fig 10:**
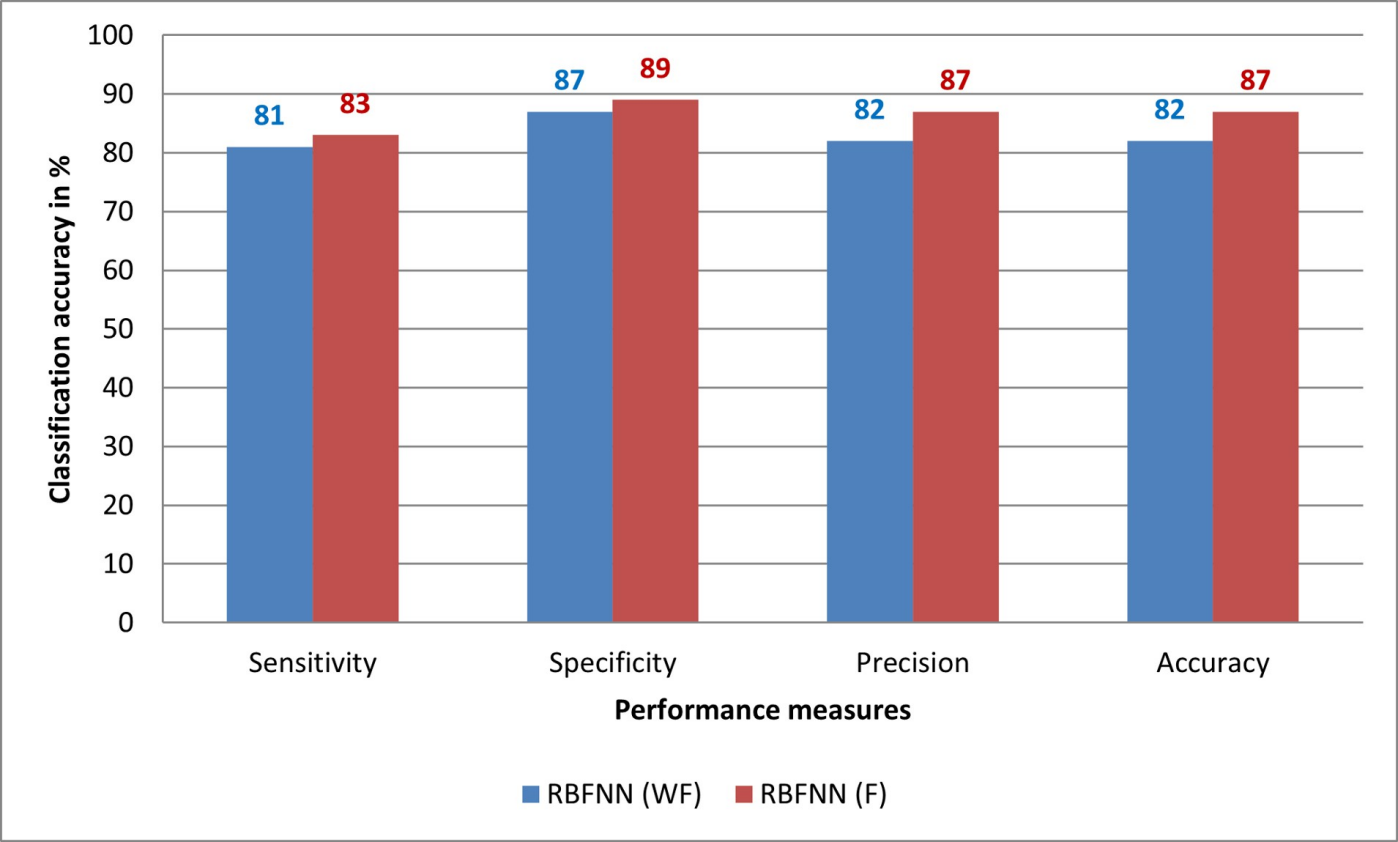
Performance analysis of RBFNN classifier in leaf disease detection.

Here, FP refers to the false positive, TP refers the true positive, TN refers the true negative, and FN refers the false negative. In Fig 10 shows the performance analysis of Radial Basis Function based Neural Network based on with fusion and without fusion.

**Fig 11:**
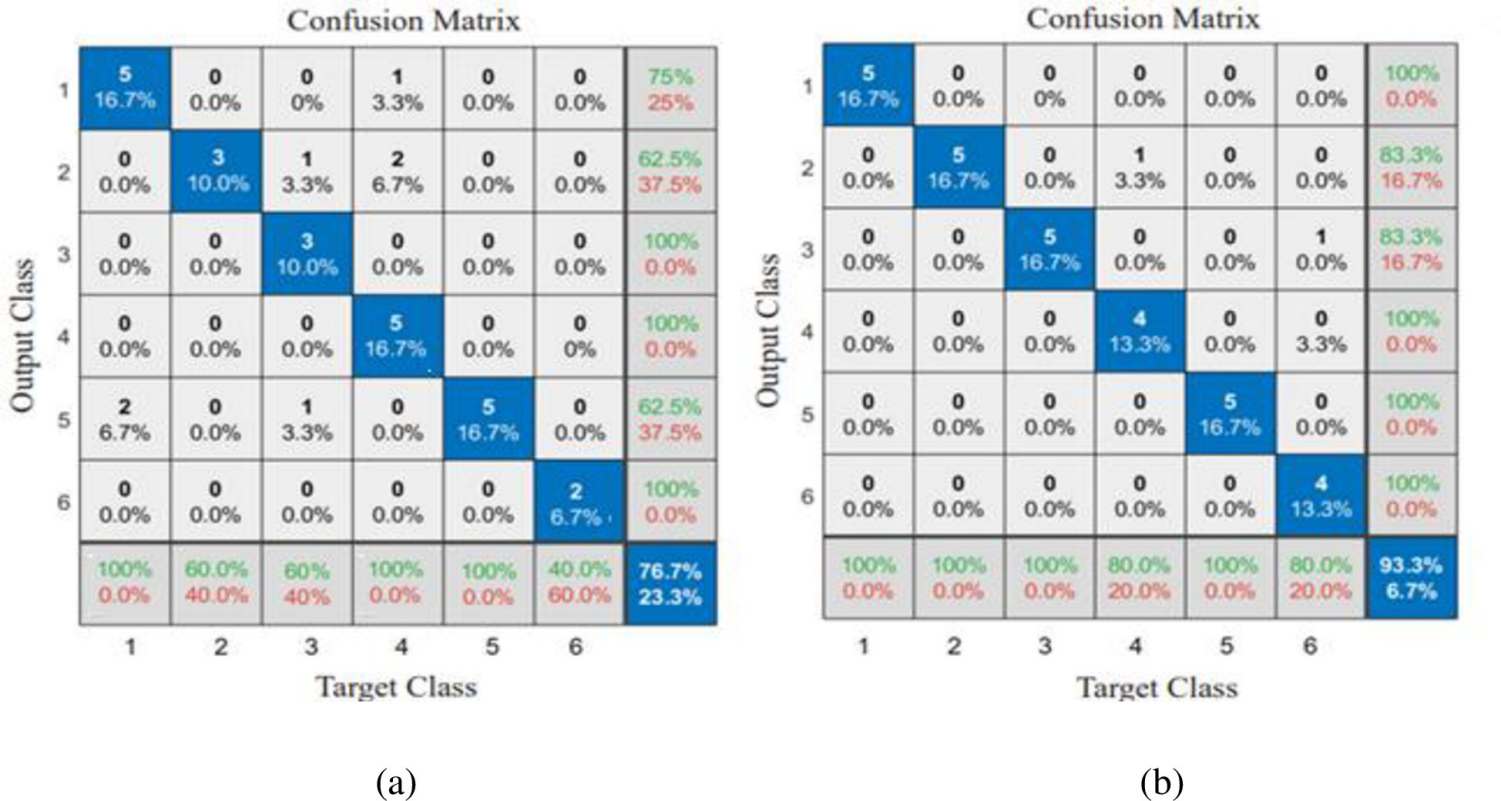
Confusion Matrix (a) DCNN without fusion (b) DCNN with fusion.

As shown in Fig 11, the performance analysis for the Deep Convolutional Neural Network is greater than the existing Radial Basis Function Neural Network by all the performance measures. Also, Specificity, Sensitivity, and precision for FBFNN and DCNN are compared with fusion and without fusion. The DCNN with fusion attained the maximum accuracy of 93.30% when compared to the DCNN without fusion (76.67%).

The validation accuracy of DCNN was computed using confusion matrix as shown in Fig 11. This figure showed that Alternaria melongenea disease primarily impacted performance. There was a 76.7% validation accuracy for RBFNNs and 93.3% for DCNNs, respectively. A DCNN was found to produce better results, thus improving the learning of leaf features. The symptoms of Alternaria melongenea and Tobacco Mosaic Virus do not have clear patterns and also have a powdery appearance on the leaf surface due to pests or insects. It may be difficult to detect Altermaria melongena and Tobacco Mosaic Virus because of the discoloration of the leaves.

A comparison of two classifiers in detecting the leaf diseases with and without feature fusion is given in Table 8.

**Table 8:** Performance comparison between RBFNN and DCNN in leaf disease detection.

Classifiers	Without Fusion	With Fusion
RBFNN	82.00%	87.00%
DCNN	76.70%	93.30%

Though the present work gives a promising result on brinjal leaf disease classification, it also has the following limitations:

- The plant village dataset has a large number of images collected in a controlled environment and has been widely used for leaf disease detection. Although this study used a limited number of images (1500 images for six classes), the number of images may not be sufficient to develop a more robust and generalized leaf disease detection system.
- It is also imperative to note that the size of the images in each class is not the same. This may lead to data imbalance issues when developing the deep learning model.
- Our own dataset was used as the basis for developing the brinjal leaf disease detection model. Thus, we are severely limited in our ability to benchmark our experimental results against the results of earlier work in this field.

Our ongoing and future research will focus on developing a more robust and efficient system to detect brinjal leaf disease.

- By considering data collection environmental factors such as illumination, background effects, and weather, we will collect more images for each class in brinjal leaves and with different types of diseases to strengthen our dataset and develop a more robust AI model for leaf disease detection.
- In order to avoid any effects due to the data imbalance in the AI model, we will utilize data augmentation methods to balance the dataset for each class.
- To increase the robustness of our proposed leaf disease detection system, we will train our dataset with different types of Deep Neural Networks (DNNs) and test the model with universal (open source) datasets.

## 5. Conclusions

This study presents a method for detecting diseases in plants by combining image processing techniques with Deep Convolutional Neural Networks as a means of detecting disease. An automated method for detecting plant diseases will be proposed in this study using these neural networks. The images are preprocessed via wiener filtering before being sent to image segmentation methods for further processing. As a result, the quality of the leaf image is enhanced, and additional segmentation techniques (Fuzzy C-means clustering and Expectation maximization) are used to isolate the defective portions. Discrete Sherlet Transforms are used to extract color, texture, and structural information from segmented images. These features were classified using DCNN and RBFNN to identify leaf diseases. Furthermore, compared to RBFNN, the proposed eight-layer DCNN is more accurate, sensitive, and specific at identifying leaf diseases. Hence, it is possible to develop mobile apps and pocket-sized devices that are able to analyze vast areas without requiring expert knowledge within a short period of time in the future by applying the proposed methodology.
